# Gestational Dietary Protein Is Associated with Sex Specific Decrease in Blood Flow, Fetal Heart Growth and Post-Natal Blood Pressure of Progeny

**DOI:** 10.1371/journal.pone.0125694

**Published:** 2015-04-27

**Authors:** Juan H. Hernandez-Medrano, Katrina J. Copping, Andrew Hoare, Wendela Wapanaar, Rosalie Grivell, Tim Kuchel, Giuliana Miguel-Pacheco, I. Caroline McMillen, Raymond J. Rodgers, Viv E. A. Perry

**Affiliations:** 1 School of Veterinary and Medical Science, University of Nottingham, Sutton Bonington, United Kingdom; 2 School of Paediatrics and Reproductive Health Robinson Institute, University of Adelaide, Adelaide, Australia; 3 South East Vets, Mt Gambier, South Australia, Australia; 4 The Chancellory, University of Newcastle, Newcastle, New South Wales, Australia; 5 South Australian Health and Medical Research Institute, Adelaide, Australia; 6 Department of Perinatal Medicine, The Womens and Childrens Hospital, North Adelaide, Australia; University of Barcelona, SPAIN

## Abstract

**Study Overview:**

The incidence of adverse pregnancy outcomes is higher in pregnancies where the fetus is male. Sex specific differences in feto-placental perfusion indices identified by Doppler assessment have recently been associated with placental insufficiency and fetal growth restriction. This study aims to investigate sex specific differences in placental perfusion and to correlate these changes with fetal growth. It represents the largest comprehensive study under field conditions of uterine hemodynamics in a monotocous species, with a similar long gestation period to the human. Primiparous 14mo heifers in Australia (n=360) and UK (n=180) were either individually or group fed, respectively, diets with differing protein content (18, 14, 10 or 7% crude protein (CP)) from 60d prior to 98 days post conception (dpc). Fetuses and placentae were excised at 98dpc (n = 48). Fetal development an median uterine artery blood flow were assessed monthly from 36dpc until term using B-mode and Doppler ultrasonography. MUA blood flow to the male feto-placental unit increased in early pregnancy associated with increased fetal growth. Protein restriction before and shortly after conception (-60d up to 23dpc) increased MUA diameter and indices of velocity during late pregnancy, reduced fetal heart weight in the female fetus and increased heart rate at birth, but decreased systolic blood pressure at six months of age.

**Conclusion and Significance:**

Sex specific differences both in feto-placental Doppler perfusion indices and response of these indices to dietary perturbations were observed. Further, maternal diet affected development of fetal cardiovascular system associated with altered fetal haemodynamics *in utero*, with such effects having a sex bias. The results from this study provide further insight into the gender specific circulatory differences present in the fetal period and developing cardiovascular system.

## Introduction

It has been established that the male fetus is more susceptible to placental insufficiency and adverse pregnancy outcomes [1,2]. Increased median uterine artery resistance indices have been used in human medicine to identify placental insufficiency. These techniques have been adapted for use in the bovine [3,4] where it was shown that characteristic variations in the median uterine artery (MUA) blood flow could be observed over pregnancy. Moreover, these blood flow volume (BFV) measures were correlated with birth weight of calves.

Dietary restriction in early gestation affects fetal growth from 36dpc to the end of the first trimester [5–7] and may affect postnatal reproduction and production traits in cattle progeny [8–13]. Critical windows exist during oocyte, embryo and fetal development where decreased maternal dietary protein may reset early cell lineages [14]. In sheep maternal nutrition affects oocytes number, quality [15] and embryo survival [16]. In cattle sex specific differences are seen in embryo glucose uptake [17] DNA methylation pattern [18], expression of key developmental genes [19] and fetal and placental perfusion [1,16]. Intriguingly, neonatal and fetal death in cattle is increased if the fetus is male [20,21], as reported in other mammalian species [22,23].

The aim of this study is to further evaluate the critical windows during development where dietary protein may alter fetal development and subsequently impact upon lifetime health and production. This paper focuses on the effects of protein restriction during specific windows of development known to affect growth of the fetus; preconception [24] periconception (including implantation) [7] and first trimester [20], upon blood flow to the fetus and the developing cardiovascular system. We hypothesise that low maternal protein during the peri-conception period and the first trimester will increase fetal blood flow and cardiovascular development in a gender dependent manner.

## Materials and Methods

### Ethics statement

This project was approved by the University of South Australia IMVS Animal Ethics Committee (Approval number 18/11; Experiment 1) and the United Kingdom’s Animals (Scientific Procedures) Act 1986 (Experiment 2), respectively.

### Experiment 1 (Expt 1) Peri-conception and Post-implantation

The purpose of this experiment was to evaluate the impact of dietary protein during the peri-conception (PERI; day -60 to day 23 (implantation being 18-22dpc); insemination day = conception, day 0) and post-implantation (POST; day 23 to day 98) periods in heifers. This study was carried out at Tungali, South Australia (Lat. 34°30’9.2” South, Long. 139°18’8.3” East) and full details of materials and methods have been published previously [7]. Briefly, primiparous *Bos taurus* x *Bos indicus* heifers (n = 360) were selected and acclimatized for 60 days prior to the start of the experiment. Heifers that did not eat well in individual stalls were removed from the study. After acclimatisation, heifers were split into 4 groups based on a two-by-two crossover factorial design with conception stage (PERI) or post-implantation (POST) and dietary crude protein (CP) content (14% or 7%CP; reflecting pasture conditions in Australia) as main factors. Heifers were randomly assigned according to bodyweight (BW) to either high (H; 14%CP, n = 177; 345.5 ±26.9kg BW (mean ±S.E.M.)) or low CP diet (L; 7%CP, n = 173; 343.1 ±25.5kg BW) sixty days prior to conception. At 23 days post-conception (dpc), L and H groups were split into two further groups based on %CP content (post-implantation H and L groups) to give the crossover design resulting in 4 groups as follows: HH group (PERI 14%CP and POST 14%CP; n = 88); HL group (PERI 14%CP and POST 7%CP; n = 89); LH group (PERI 7%CP and POST 14%CP; n = 87) and LL group (PERI 7% and POST 7%; n = 86). Diets used in the experiment varied in crude protein content but were otherwise isocaloric and supplemented with a commercial vitamin and mineral preparation. The pellet portion of the diet was fed individually to the heifers daily in stalls with straw *ad libitum* in pens. At the end of the first trimester of gestation (98 dpc), all heifers were fed a similar maintenance diet (12%CP) until parturition. Detailed composition of the heifer rations was previously published [7] and a brief nutrient content is presented in Table 1.

**Table 1:** Nutrient content of heifer rations used in Experiment 1 (Australian n = 360; PERIconception and POSTimplantation) and Experiment 2 (UK, n = 188; PREconception and POSTconception) based on a dry matter basis.

	Experiment 1 * (Australia)				Experiment 2 † (United Kingdom)				
DIET	PERIconception		POSTimplantation		PREconception			POSTconception^+^	
	Low	High	Low	High	Low	High	Low		High
**Dry Matter (%)**	7.2	8.3	11.8	12.3	13.5	13.1	28.7		28.0
**Protein (%)**	8.6	14.2	7.4	12.1	10.2	18.1	10.2		14.0
**ME (MJ)**	6.3	7.1	9.8	10.2	10.7	10.9	9.9		10.0
**Calcium (Ca; g)**	22	26	37	38	50	70	-		-
**Phosphorus (P; g)**	17	17	21	21	40	40	-		-
**Ca: P ratio**	1.3	1.5	1.8	1.8	1.4	1.8	-		-

* Experiment 1 treatments: PERIconception diet, from 60 days prior to conception until 23 days post conception (dpc); and POSTimplantation diet from 23dpc to 98dpc. Low: 7% crude protein; High: 14% crude protein.

† Experiment 2 treatments: PREconception diet, from 60 days before up to conception (Low: 10%CP and High: 18%CP); and POSTconception diet from conception until 90 days post conception (Low: 10%CP and High: 14%CP).

^+^ Experiment 2 POSTconception diet calcium and phosphorus were not analysed.

### Synchronization Protocol

Oestrous cycles were synchronized 50 days after initiation of diet treatment using intravaginal progesterone releasing devices (n = 2; progesterone 1.56g, CUE-MATE, Bioniche Animal Health, Bayer Australia Ltd, Pymble, Australia) inserted sequentially on day -10, relative to artificial insemination (AI; day 0), with simultaneous intramuscular (i.m.) injection of 2mg oestradiol benzoate (Bomerol, Bayer Australia Ltd, Pymble, Australia). On day -2 intravaginal devices were removed and heifers received i.m. injections of prostanglandin 2 alpha (2ml; Ovuprost) and equine chorionic gonadotrophin (300 IU; eCG; Pregnecol). Twenty-four hours after device removal, heifers were injected i.m. with 1mg oestradiol benzoate and inseminated 24 hours later (day 0 = conception day) with frozen semen from a single bull (*Bos indicus x Bos taurus*) with known estimated breeding values (EBVs) for growth and low birth weight.

### Experiment 2 (Expt 2) Pre- and post-conception

The aim of this experiment was to further study the effects of dietary protein during differing intervention periods and at levels representative of UK commercial beef cattle protein intake. Dietary intervention occurred from day -60 to conception (AI day = conception, day 0) or from conception to 90dpc. Eighty-eight primiparous, 10–14 month-old *Bos taurus* heifers in two locations (North Yorkshire: Lat. 54°23’41.3” North, Long. 1°18’11.6” West; and Buckinghamshire: Lat. 51°34’15.7” North, Long. 0°38’32.2” West) were selected and allocated to two regimens during the preconception period (PRE, n = 88) from -60d to conception of either high crude protein (18%CP) or low dietary protein (10%CP). A further group (n = 100, POST; Northumberland, UK: 55°22’11.6” North, 1°45’04.8” West) were allocated to two dietary regimens from conception to 90dpc fed either 10%CP (Low) or 14% CP (High). This resulted in 4 independent groups: pre-conception high (PRE H, n = 44; 347.2 ±9.4kg BW), pre-conception low (PRE L, n = 44; 355.5 ±10.8kg BW), post-conception high (POST H, n = 51; 392.6 ±3.8kg BW) and post-conception low (POST L, n = 49; 393.9 ±3.6kg BW). Heifers in this experiment were group-fed the corresponding diets. After the end of the dietary intervention, all animals were run together on each individual farm under similar diet and management practices for the remainder of the pregnancy.

### Synchronization Protocol

Oestrous synchronisation was carried out by a commercial breeding company following a 5-day synchronization protocol for beef heifers reported previously by Bridges *et al*.[25]. Similarly to experiment 1, following synchronisation heifers were inseminated using semen from a single bull with known EBVs for growth and low birth weight on each farm.

### Heifer and Fetal Measurements

Fetal growth was measured using trans-rectal ultrasound (probe 5–12.5; Sonosite M-Turbo, Sonosite Inc., Bothell, Washington, USA) at 35 and 60 dpc. Simultaneously, fetal crown-rump length (CRL; 35dpc), biparietal diameter (BPD; 60dpc) and sex (60dpc) were recorded for all pregnant heifers. Crown-rump length (CRL) was measured from a lateral view of the fetus from the tip of the nose to the base of the tail, while biparietal diameter (BPD) was measured from a dorso-ventral view of the cranium and perpendicular to the sagittal crest as the widest span between the most lateral parts of the parietal bone [5]. After 60dpc, empty heifers were withdrawn from the experiment.

Transrectal pulsed B mode Doppler sonography (Sonosite M-Turbo, Sonosite Inc., Bothell, Washington, USA) of the MUA (as described previously [4]) was performed at 120, 150, 180 and 210 dpc. Briefly, in longitudinal color Doppler section of the artery, blood-flow waveforms were measured at an interrogation angle between Doppler ultrasound beam and flow direction from 45° to 60°, with the volume sample mark located in the middle of the vessel. Evaluations of the MUA diameter were done in a cross-section of the vessel just forward of the external iliac vein [26]. Doppler indices, such as resistance index (RI), pulsatility index (PI) and timed average maximum velocity (TAMV) were recorded. Total blood flow volume (BFV) to the pregnant uterus was estimated following the equations presented by Herzog *et al*.[4]. Time constraints considering the number of heifers to be scanned dictated that only the ipsilateral MUA was scanned over two days. The relationship between the two arteries in blood flow parameters of resistance, velocity and volume have previously been reported [26], with resistance decreasing and velocity, volume and diameter increasing in the ipsilateral side at an increased rate from the 4^th^ month of pregnancy.

At 98dpc, a subset of heifers (HH = 12; HL = 15; LH = 10; LL = 9) in Expt 1 were killed at a commercial abattoir. The pregnant uterus from each heifer was collected and the fetus excised, blood sampled, measured (CRL and BPD, as previously described; [7]) and weighed. Fetal sex was recorded and tissue samples (i.e. heart) weighed and collected. The remaining heifers in Exp 1 were allowed to calve and postnatal arterial blood pressure measured as describe in the following section.

### Postnatal Arterial Blood Pressure

In Experiment 1, arterial blood pressure was measured to determine the long-term effects of maternal under-nutrition during gestation on the cardiovascular system in the offspring. This was carried out using the tail-cuff system previously described and validated for calves [13] at 6, 8 and 10 months of age (200, 250 and 350d, respectively), using a non-invasive blood pressure monitor (Cardell Veterinary Monitor 9401BP, SHARN Veterinary). Briefly, the measurement was taken by the same operator on each occasion whilst the calf was restrained quietly in a crush. At least five consecutive measurements were taken for each calf. Only measures where three of the readings ranged by <10 mmHg were used to obtain a mean [13]. Those with greater variation were discarded.

### Statistical Analysis

All data was analysed using STATA software (STATA/IC for Windows; StataCorp LP, College Station, Texas, USA). Data was checked for normality and transformed if required. Data regarding fetal measurements (CRL and BPD) was analysed using factorial ANOVA with diet (L or H), conception stage (PRE, PERI- POSTconception and POSTimplantation) and fetal sex as main factors, with interactions between these being explored. Experiment 1 and 2 were analysed separately as dietary treatment and intervention periods differed. Additionally, for experiment 2 a farm factor was used as a blocking factor. As heifer is considered the experimental unit, repeated measurements ANOVA with Greenhouse-Geisser epsilon adjustment for the within-subject variance was used when comparing Doppler measurements. Data is presented as the mean values of the raw data ± standard error of the mean (S.E.M) unless stated otherwise. After comparison of means (hypothesis tests), a p-value ≤ 0.05 was considered as statistical significant and a P-value ≤ 0.10 considered as a tendency.

## Results

### Uterine Artery Blood Flow

**Fig 1:**
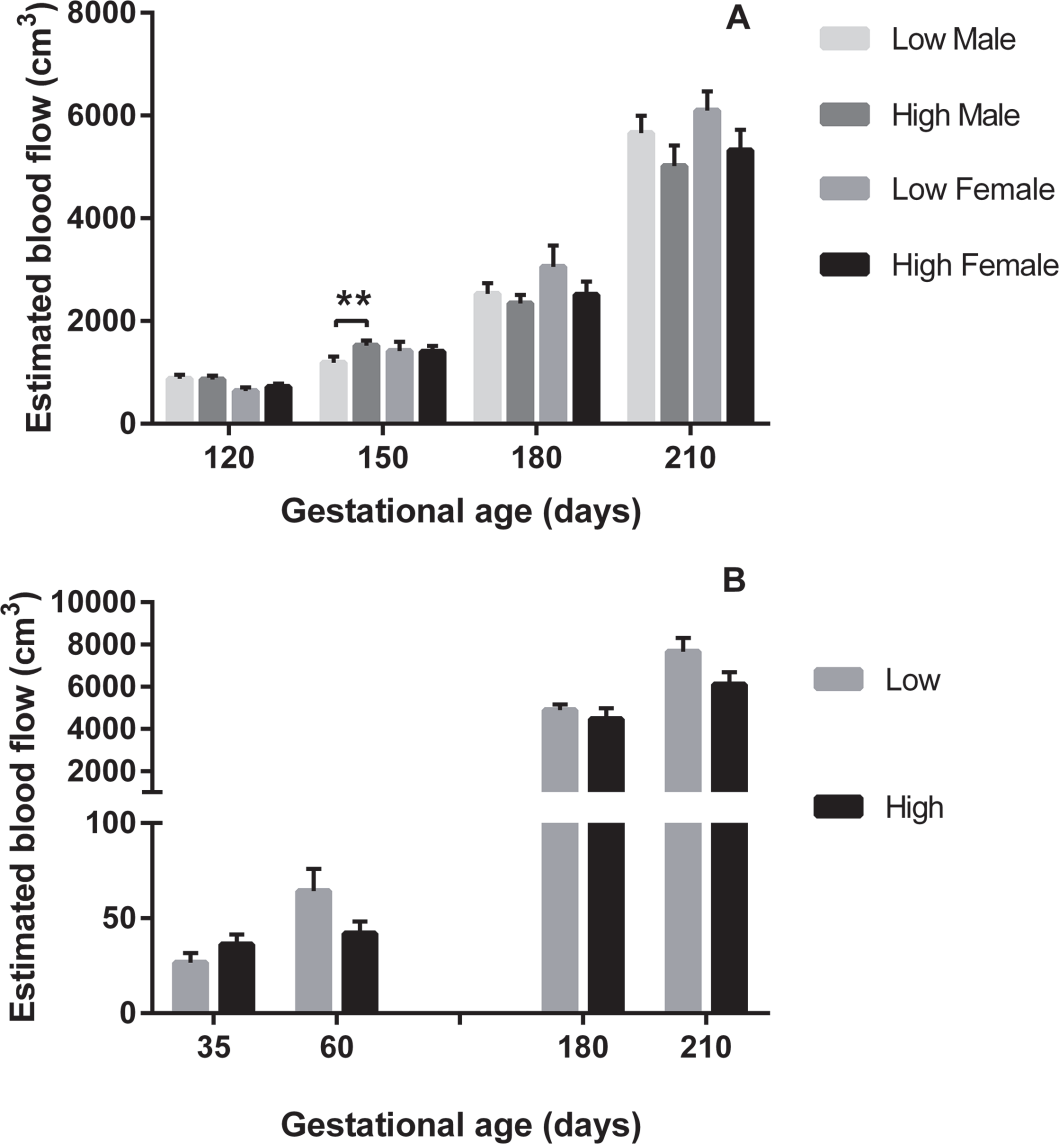
Blood Flow Volume according to sex and PERI and PREconception diet. Mean (±SEM) estimated blood flow volume (cm^3^) in heifers carrying male (M) or female (F) fetuses by treatment diet in A) Experiment 1 (Low 7%CP, M n = 20 and F n = 8; and High 14%CP,M n = 25 and F n = 11, from 60d prior to conception to 23dpc) and in B) Experiment 2 (Low 10%CP, M n = 13 and F n = 3; and High 18%CP, M n = 7 and F n = 11 from -60d to conception); ** denotes P≤0.01.

**Fig 2:**
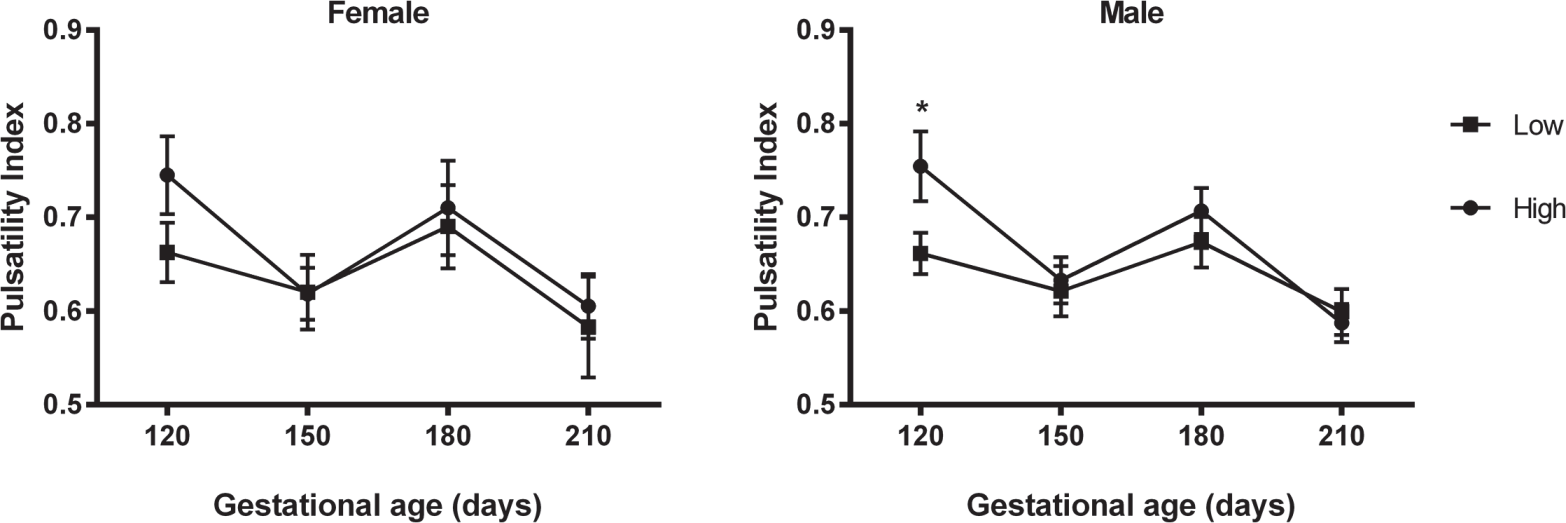
Median Uterine Artery diameter according to PERI and PREconception diet. Mean (±SEM) Median Uterine Artery (MUA) diameter (mm) in heifers carrying male (M) and female (F) fetuses according to A) PERIconception diet Low (7%CP; M n = 20 and F n = 8) and High protein (14%CP; M n = 25 and F n = 11) from 60d prior to conception to 23dpc in Experiment 1 and B) PREconception diet Low (10%CP; M n = 13 and F n = 3) and High protein (18%CP; m n = 7 and F n = 11) from -60d to conception in Experiment 2.; ** denotes P≤0.01.

**Fig 3:**
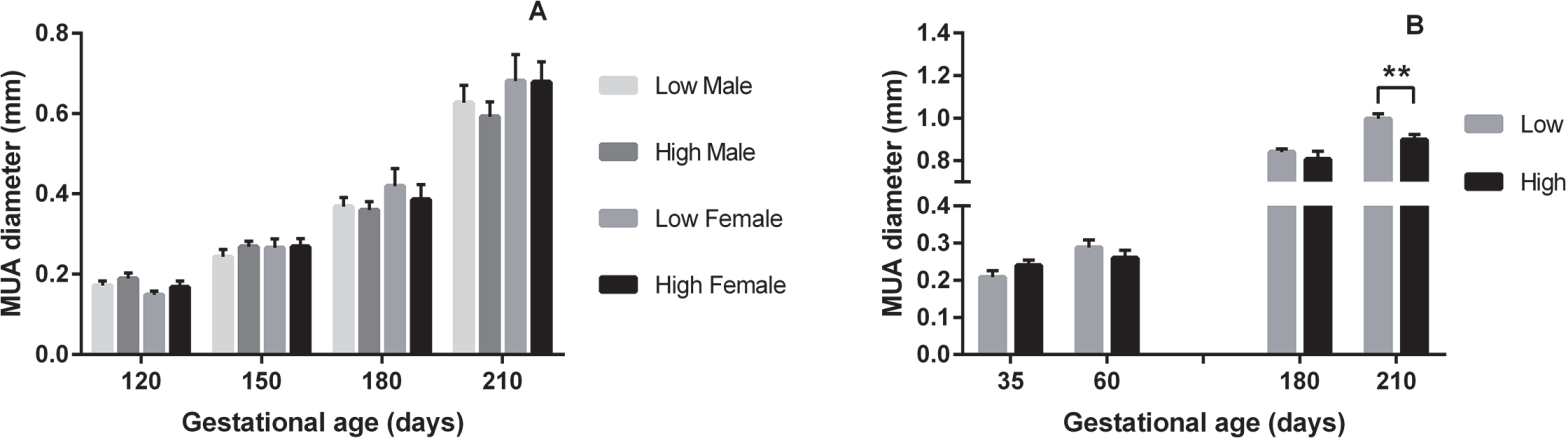
Pulsatility Index according to PERIconception diet. Pulsatility Index (mean ±SEM) by PERIconception diet (■ Low 7%CP and ● High 14%CP) from 60d prior to conception to 23dpc in Experiment 1 in male (Low n = 20 and High n = 25) and female fetuses (Low n = 8 and High n = 11). * denotes P≤0.05.

Blood flow volume (BFV; Fig 1) increased over gestation in heifers in all experimental treatments (P = 0.01) in association with reduced PI (P<0.001) and increased MUA diameter (P = 0.01; Fig 2). In Expt 1, BFV increased over gestation (P<0.001), this effect being influenced by sex (P = 0.01) and PERIconception diet (P = 0.005; Fig 1A). Those heifers with a male feto-placental unit at 120dpc had significantly greater BFV (P = 0.03) compared to those with a female. This effect on BFV was associated with low PERIconception protein reducing PI in all heifers at 120dpc compared to those on the high PERIconception diet (P = 0.02; Fig 3). Moreover heifers in the low PERIconception diet carrying male fetuses had an increased PI (P = 0.05) and a reduced BFV at 150dpc (P = 0.02), compared to those that received the high PERIconception diet.

**Fig 4:**
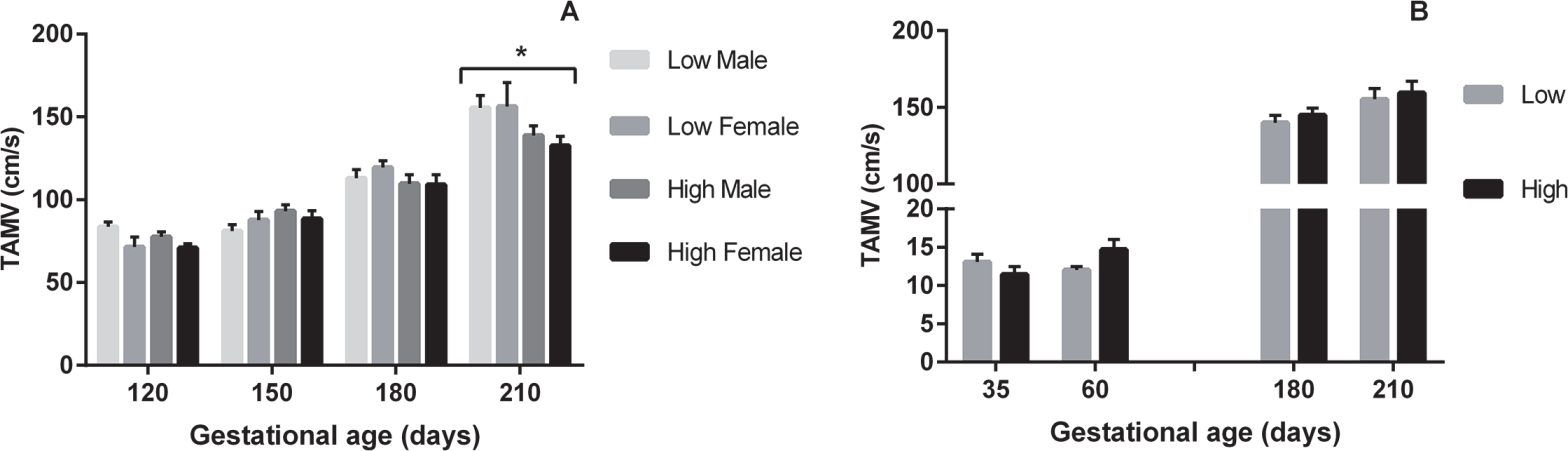
Time Average Maximum Velocity according to PERI and PREconception diet. Mean (±SEM) Time Average Maximum Velocity (TAMV; cm/s) in heifers carrying male (M) and female (F) fetuses according to A) PERIconception diet Low (7%CP; M n = 20 and F n = 8) and High (14%CP; M n = 25 and F n = 11) from 60d prior to conception to 23dpc in Experiment 1 and B) PREconception diet Low (10%CP; M n = 13 and F n = 3) and High (18%CP; M n = 7 and F n = 11) from -60d to conception in Experiment 2; * denotes P≤0.05.

Similarly TAMV was affected by sex (P = 0.03) and diet (P = 0.05). At 120dpc TAMV was increased in those heifers carrying a male rather than a female fetus (P = 0.03; Fig 4). At 150dpc, TAMV increased in heifers receiving the low compared to high PERIconception protein diet (P = 0.05). At 210dpc, heifers fed the low PERIconception or POSTimplantation protein diet had increased TAMV (P = 0.03; Fig 4A) compared to those fed the high diet during these periods.

**Fig 5:**
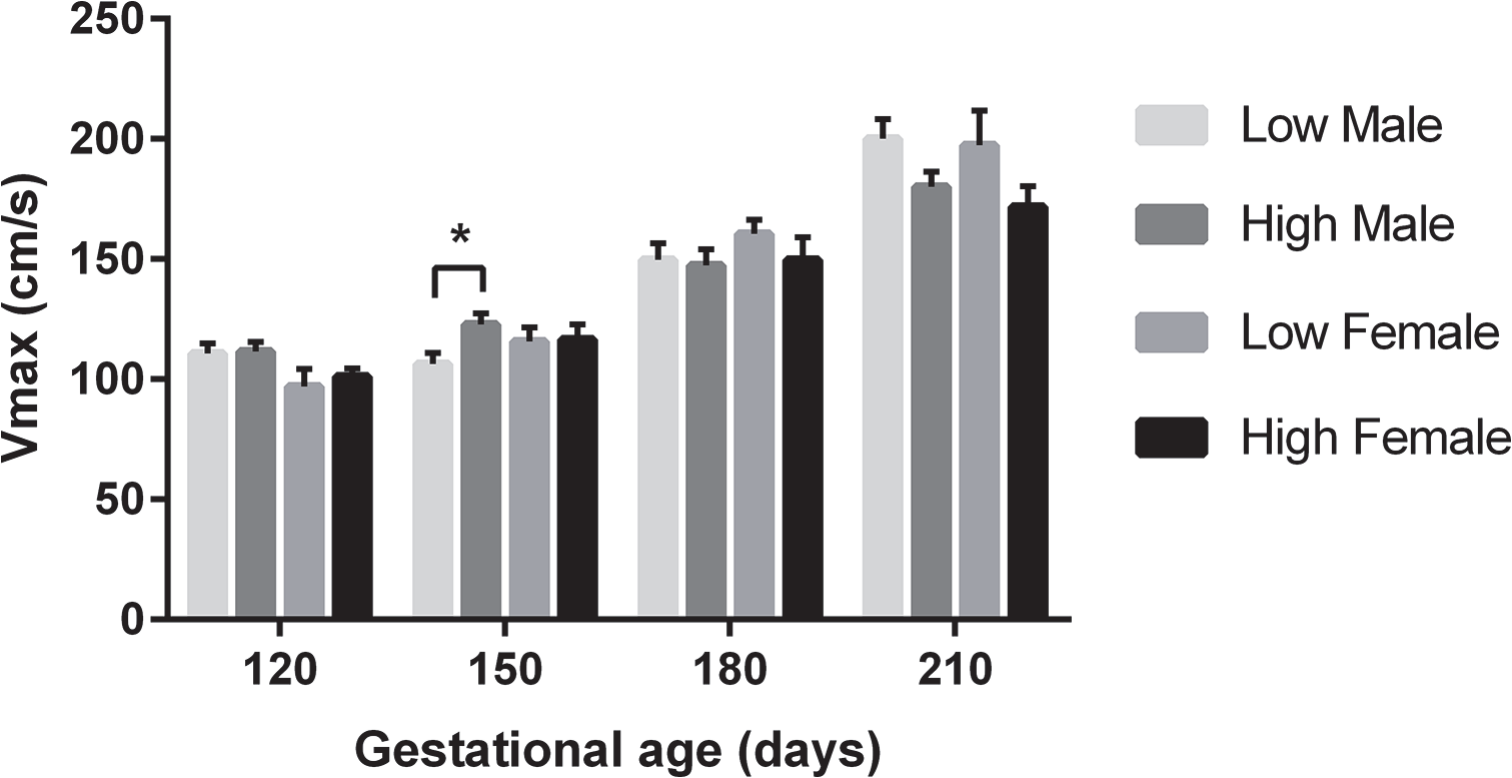
Maximal Velocity according to PERIconception diet. Mean (±SEM) Vmax (maximal velocity; cm/s) in heifers carrying male (M) and female (F) fetuses according to PERIconception diet Low (7%CP; M n = 20 and F n = 8) and High (14%CP; M n = 25 and F n = 11) from 60d prior to conception to 23dpc in Experiment 1. ** denotes P≤0.01.

Maximal velocity (Vmax) was affected similarly to TAMV. At 150dpc, heifers with a male fetus fed the low protein PERIconception diet had a lower Vmax than heifers fed the high diet (P = 0.01; Fig 5). At 210dpc, heifers with a male fetus fed the low PERI and POSTimplantation diet had a higher Vmax measurement than heifers that received the high PERI and POSTimplantation diet (P = 0.03).

**Fig 6:**
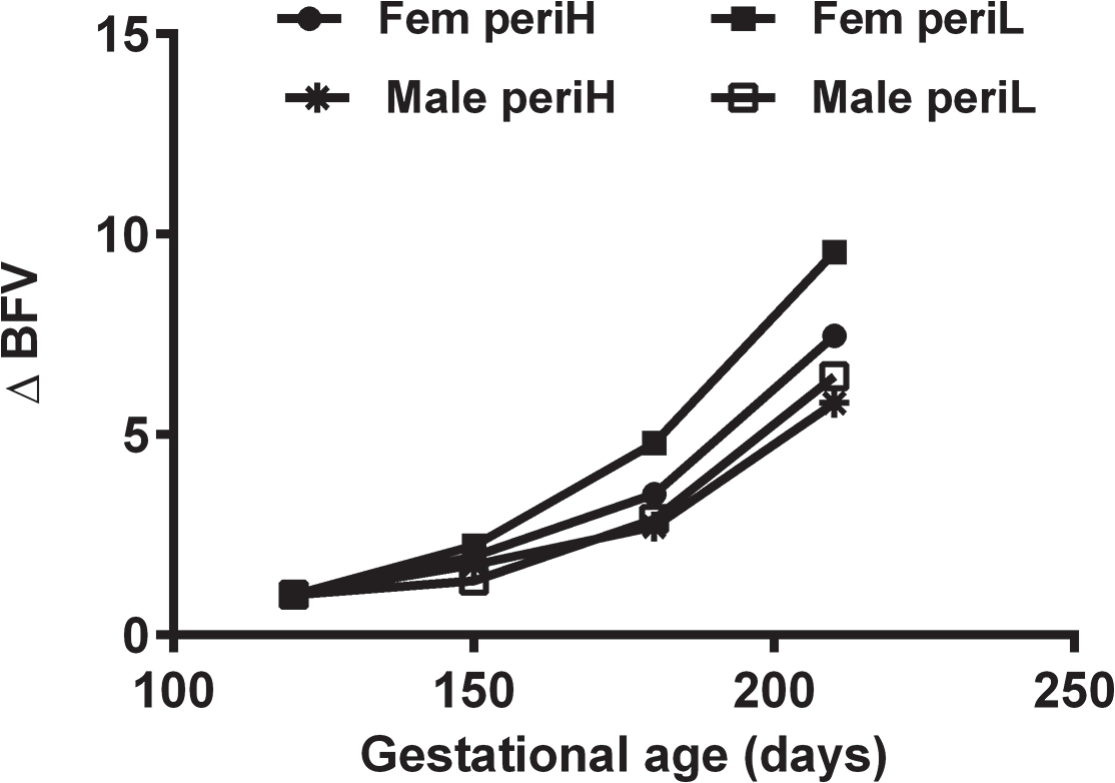
Mean Blood Flow Volume change according to sex and PERIconception diet. Mean Blood Flow Volume change (BFV ∆) over time (as a ratio of the initial measure at 120dpc) of heifers according to fetal sex and PERIconception diet (Male: PERI High *, n = 25, and PERI Low □, n = 20; female: PERI High ●, n = 11, and PERI Low ■, n = 8). PERIconception diet was given from day 60 prior to conception to day 23 post-conception in Experiment 1.

In combination PERIconception low protein diet increased blood flow to fetus over gestation by reducing PI (resistance) and increasing TAMV (velocity; Fig 6).

In Expt 2, there were lower numbers of heifers per treatment group (n = 40 in the PREconception dietary treatments and n = 50 in the POSTconception treatments). There was a trend for the low PREconception protein diet to increase BFV (P = 0.06; Fig 1B) and TAMV (p = 0.06) at both 36 and 60dpc and at 180 and 210dpc (P = 0.08; Fig 4B). At 210dpc MUA diameter was increased in those heifers that received the low PREconception diet (P = 0.01; Fig 2). The low protein POSTconception diet increased MUA diameter (P = 0.04) at 60dpc associated with a trend to increased BFV (P = 0.09).

### Blood Flow and Birth Weight

**Fig 7:**
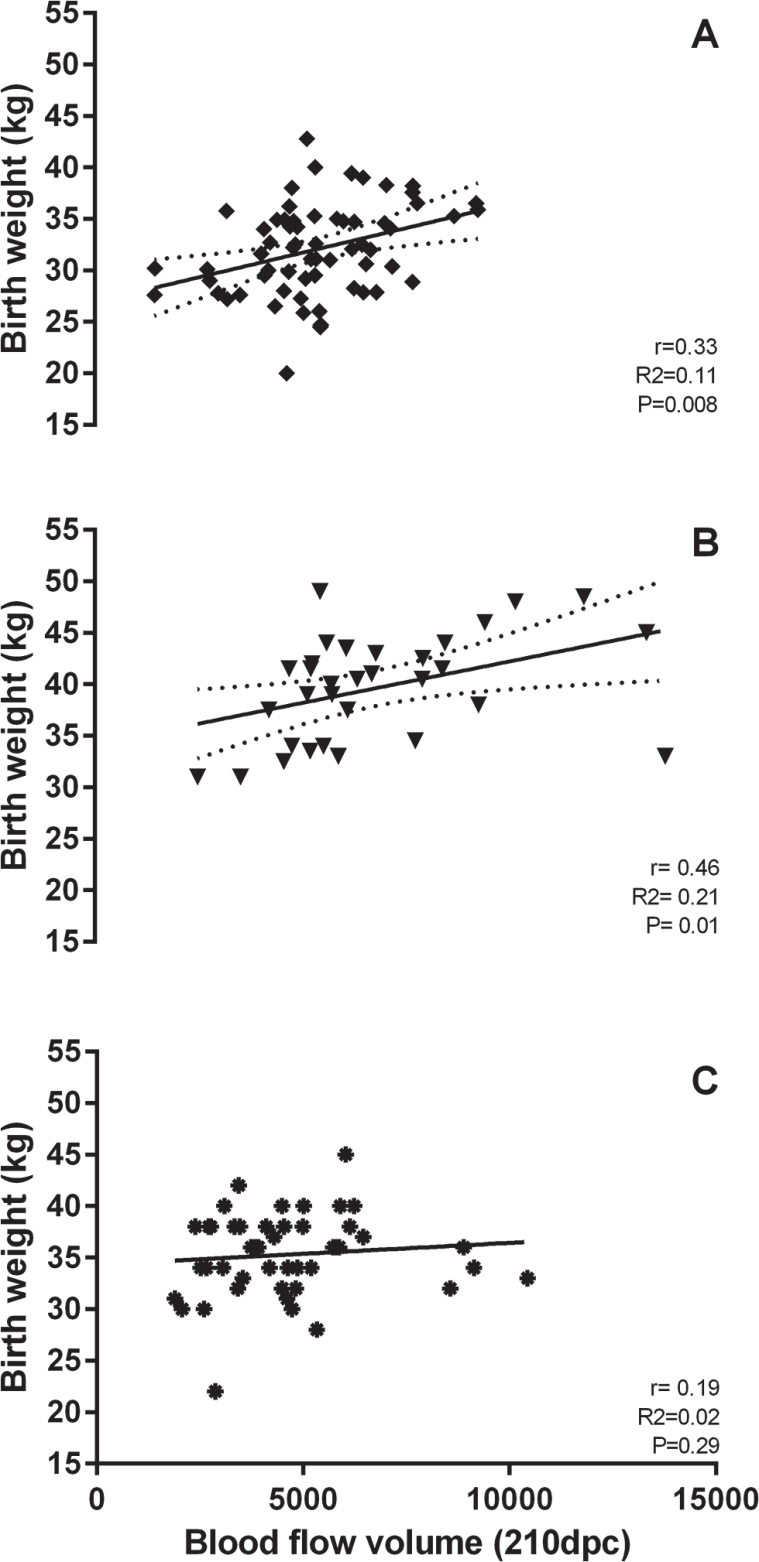
Correlation of calf birth weight and blood flow volume. Correlation (r) of calf birth weight (kg) and blood flow volume (BFV, cm^3^) at 210 dpc in calves from heifers in Experiment 1 (A, PERIconception diet and B, POSTimplantation diet) and Experiment 2 (C, PREconception diet). Heifers in Experiment 1 received a Low (7%CP, n = 28) or High (14%CP, n = 36) protein diets either from 60 days prior to conception (conception = insemination = day 0) to 23d post-conception (dpc; PERIconception diet) and from 23dpc to 98dpc (POSTimplantation diet). In Experiment 2, heifers received a Low (10%CP, n = 16) or High (18%CP, n = 18) protein diet from 60d prior to conception (PREconception) to 90dpc.

Birth weight was not affected by dietary treatment in Expt 1 but in Expt 2 the low PREconception diet (10 vs 18% CP) increased birth weight in male calves (P = 0.03). Birth weight was positively correlated with MUA blood flow in both experiments under either PRE or PERIconception dietary regimen (P = 0.01; Fig 7). This effect however, was greater in male calves on the low PERIconception diet (r = 0.42, P = 0.004) with the opposite dietary effect in the female; that is, high PERIconception diet in the female increased the correlation between MUA blood flow and birth weight (r = 0.47, P = 0.03). In Expt 2 again it was the high PREconception diet that caused the significant correlation with birth weight in females (r = 0.57, P = 0.03) with a trend to significance in the low PREconception diet males (r = 0.52, P = 0.07). There was no significant correlation when males were subjected to a high PREconception diet or females to the low PREconception diet.

### Fetal Heart Weight (Expt 1)

**Fig 8:**
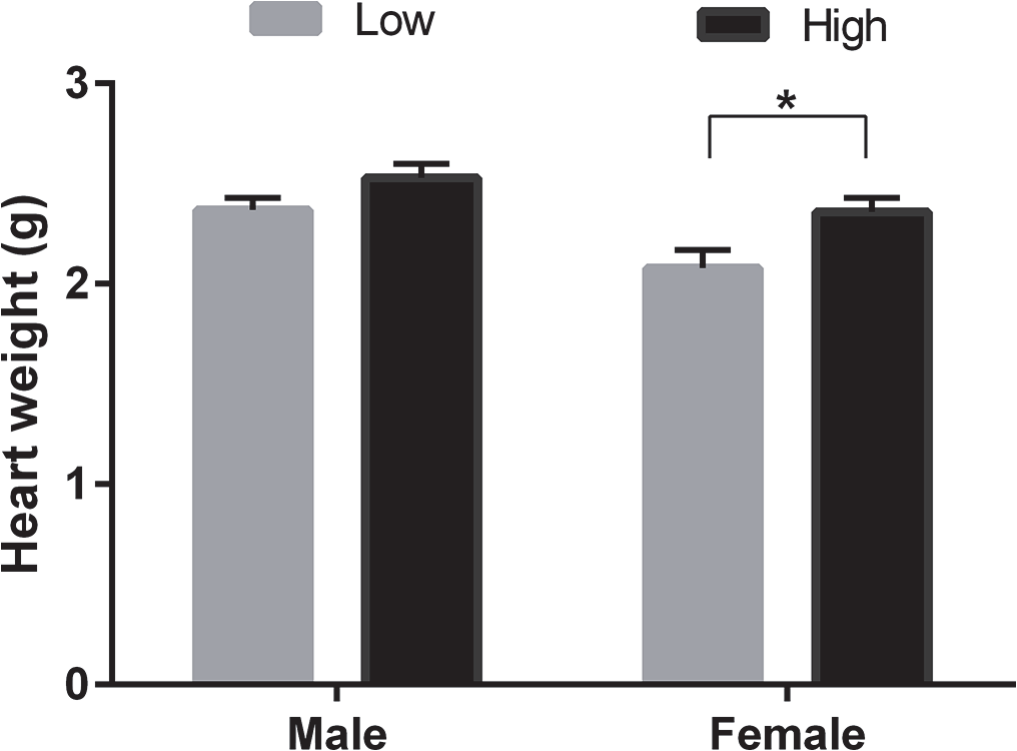
Post-mortem fetal heart weight according to PERIconception diet. Mean (±SEM) post-mortem fetal heart weight (g) in male (M) and female (F) 98-day-old fetuses from heifers fed Low (7% CP, M n = 9 and F n = 10) or High (14% CP, M n = 16 and F n = 11) PERIconception diet from day 60d prior conception to 23days post-conception.

In the excised 98dpc fetus low protein diet (7%CP) compared to high (14% CP) during the PERIconception period reduced fetal heart weight in female fetuses (P<0.05; Fig 8).

### Fetal Growth (Expt 2)

**Fig 9:**
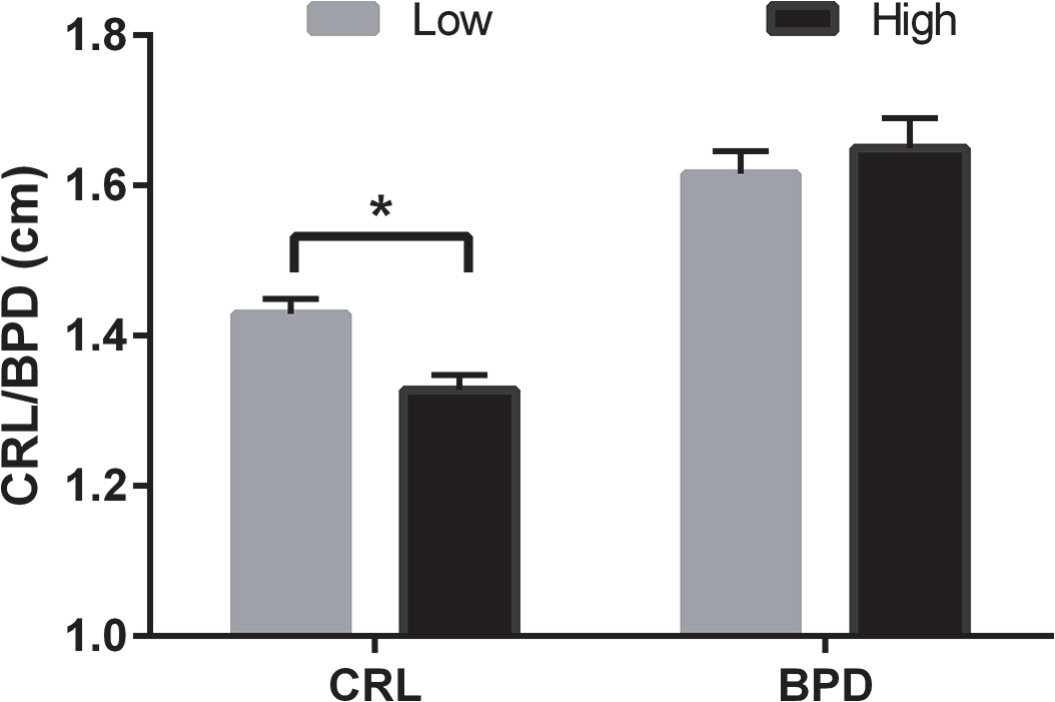
Crown-Rump length and the Biparietal Diameter at 60dpc according to POSTconception diet. Mean (±SEM) crown-rump length (CRL, cm) at 35dpc and biparietal diameter (BPD, cm) at 60dpc in pregnant heifers that received a High (14% CP; n = 25) or Low (10% CP; n = 24) protein diet during the POSTconception period (conception, day 0) to 90d post conception in Experiment 2. * indicates P<0.05.

Protein intake during the PREconception period in Experiment 2 had no effect upon fetal growth at 36 or 60dpc. Low protein (10%CP) during the first trimester (POSTconception diet) was associated with a transient increase in fetal growth as measured by CRL at 36dpc compared to the high (14%CP) diet, but this effect was not present at 60dpc (Fig 9).

### Neonatal Heart Rate

**Fig 10:**
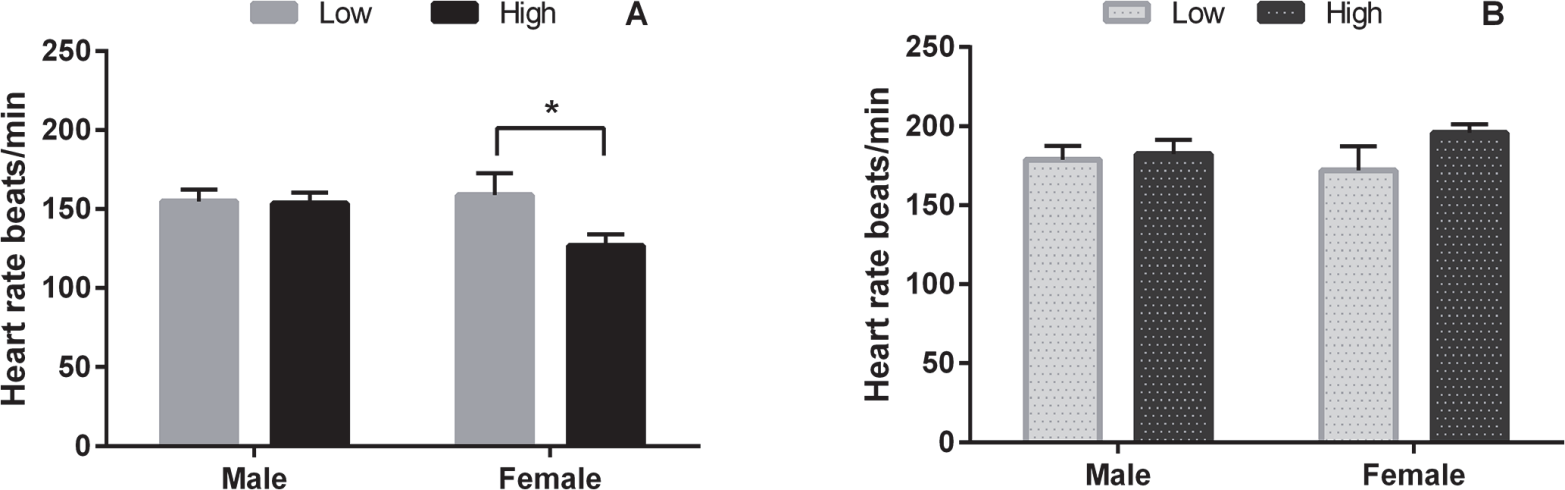
Heart rate at birth according to sex and PERI and PREconception diet. Heart rate (mean beats/min ±SEM) at birth of male and female calves from heifers fed Low (7% CP; n = 28) or High (14% CP; n = 36) PERIconception diet from day 60 prior conception to day 23dpc in experiment 1 (A); and from heifers fed Low (10% CP; n = 16) and High (18% CP; n = 18) PREconception diet from -60d to conception (day 0) diet in experiment 2 (B); * indicates P<0.05.

Low PERIconception dietary protein (7%CP) increased heart rate at birth in female offspring in Expt 1 compared to the high diet (14%CP; P<0.05). There was no effect on heart rate in the animals in Expt 2 (10% vs 18%CP; Fig 10).

### Blood Pressure in Progeny

**Fig 11:**
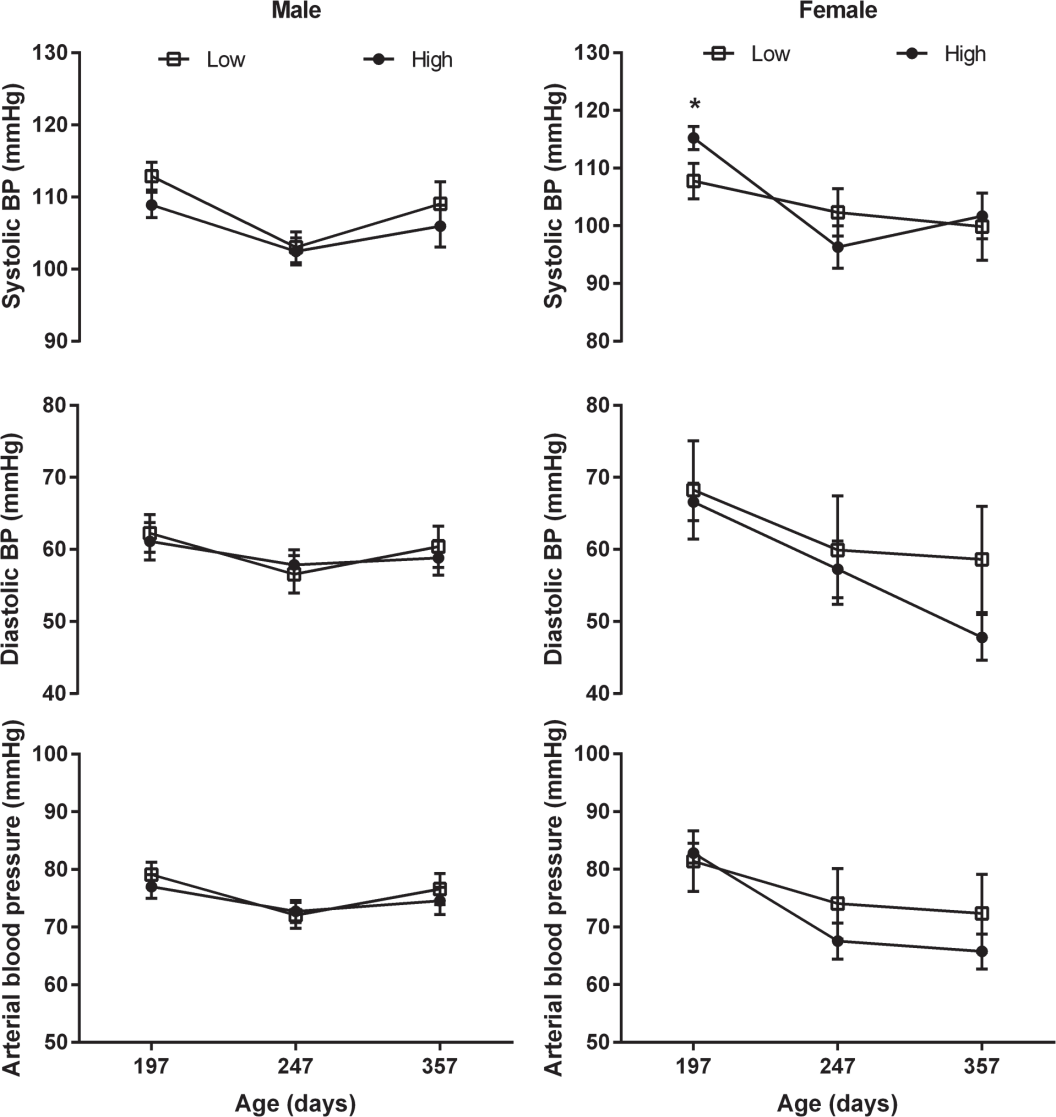
Arterial Blood Pressure according to PERIconception diet. Arterial blood pressure (mean ±SEM) in male (left) and female (right) calves of heifers fed Low (7% CP □; n = 28) or High (14% CP ●; n = 36) PERIconception diet from 60 days prior conception to 23days post conception in Experiment 1. * denotes P≤0.05.

Low protein during the PERIconception period (7% CP) decreased systolic blood pressure (P<0.05) in female progeny at six months of age with no effect at eight or ten months (Fig 11).

## Discussion

This study is the first to show sexually dimorphic effects of transient protein restriction during peri-conception upon blood flow to the placenta and the developing fetal cardiovascular system. This research represents the largest comprehensive study of uterine hemodynamics in a monotocous species, with a similar long gestation period to the human completed under field conditions.

We have recently published the first report of the enhanced sensitivity of the male fetal calf to dietary perturbation in early pregnancy, affecting growth [7] and placental function [20]. In the heifers from Expt 1, the PERIconception low protein diet at 7%CP reduced fetal growth compared with 14%CP diet with the effect being greater in the male [7]. Interestingly this effect upon growth was no longer apparent at birth. In contrast in the data presented here from Expt 2, the high protein diet (14%CP) during the first trimester of pregnancy transiently decreased fetal growth compared to the low (10%CP) diet. Furthermore, the low protein PREconception diet of 10% increased birth weight compared to the high PREconception diet of 14%.

We consider that these differing effects may be influenced by the different levels of protein and the different dietary intervention periods in the two studies. In Expt 1, the PERIconception diet did not cease until 23dpc (after implantation of the embryo) whereas in Expt 2 the PREconception diet ended at conception (i.e. AI) and the POSTconception diet started at conception. Both POSTconception diets, however, in Expt 1 and 2, continued until the end of the first trimester. We could expect this PERIconception diet, therefore, to exert a larger effect as it encapsulated both the development of the oocyte, the embryo and initial fixation to the uterus.

The protein levels in Expt 1 were also more restrictive than those in Expt 2. The low protein diet in Expt 1 allowed a daily gain of 0.2kg per day compared 0.5kg when heifers were fed the high diet (data not shown). In the UK (Expt 2), however, both the low and high protein diets allowed a gain of at least 0.7kg i.e. there was no significant difference in the weight gain of the two groups (data not shown). The low protein PREconception diet, however, yielded an increase in birth weight. This may reflect an increase in placental development, as blood flow to the placenta (at 210dpc) in these animals was also elevated compared to those on high PREconception diet with this blood flow strongly correlated with birth weight (P<0.01). Previous reports have shown contradictory effects of low protein diets during the first trimester followed by increased protein after 2^nd^ trimester on placental trophectoderm volume and cotyledon weight [27,28].

In accord with previous work in beef cattle [3] resistance indices in the MUA traced a characteristic pattern over gestation and further, similar sex specific venous flow rates were observed as in the human [1]. In Expt 1 (7% vs 14%CP) the male feto-placental unit received greater blood flow than the female at 120dpc, this being achieved by decreased resistance (PI), increased velocity (TAMV) and increased diameter of the MUA. Throughout gestation the low protein in both the PERIconception and PREconception diet enhanced blood flow to the fetus by reducing resistance and increasing volume flow in the MUA. This can be best illustrated in Fig 6 where the ratio of BFV over gestation compared to the initial measure is shown. This was associated with significantly increased velocity (TAMV) and MUA diameter (P>0.05) with no difference in resistance (PI) over the treatment groups from 150dpc.

The effects of PERIconception low protein diet upon MUA blood flow was greater in those heifers with a male fetus at both 120 and 150dpc. This is concomitant with the data presented on the correlation between blood flow and birth weight, that is when both sexes are grouped together this relationship appears consistent (P = 0.01), however when we consider this by sex the correlation between blood flow and birth weight in the male calf exists only if the heifer received a low protein diet either PREconception or PERIconception (P = 0.004). In the female calves this correlation only exists if their dams had received a high protein diet in the PERI and PREconception period (P = 0.03). This suggests that dietary perturbations may have different effects upon uterine hemodynamics and fetal development *in utero* dependent upon sex as previously reported [1,7,20].

Contrary to one recent report [29] nutrient restriction altered uterine blood flow from 120dpc to 210dpc. This apparent disparity may be caused by the larger numbers in this study, consideration here of the sex specific effects, the difference in dietary treatment (protein versus total nutrients), the use of only primiparous heifers in this study and the period of dietary perturbation. The effects of nutrient restriction upon fetal and placental growth in the primiparous compared to the mature female are significantly different [30] as the hormonal response of the mature female signals greater nutrients to the fetus than in the immature primiparous female.

The *in utero* effects upon fetal growth and blood supply in Expt 1 produced corresponding effects upon the cardiovascular system in the neonate and young offspring with neonatal heart rate being increased in the female neonate with six month old female progeny having decreased systolic blood pressure. This study supports previous work [13] that showed heart length and width was reduced by nutrient restriction in female fetus at 94dpc and that aortic diameter was increased [13]. This important study by Mossa et al. [13] showed that low protein postconception diet increased systolic pressure in the neonatal calves. PERIconception diet in the current study however decreased systolic pressure at six months of age. Interestingly, the low protein diet (7%vs 14%) significantly reduced fetal heart weight in the female compared to the male fetus at 98dpc.

The relationship between blood flow and resistance indices in the two arteries has previously been published in beef cows [26]. Due to the large number of animals in this study (n = 360 in Expt 1 and n = 188 in Expt 2), only the ipsilateral artery to the fetus was assessed by Doppler, as it is known to have a consistent relationship with the increased blood flow in the contralateral artery, although with an average four-fold increase in blood flow, compared to the contralateral artery during pregnancy [3].

In humans, when uterine artery resistance does not decrease during pregnancy there is an association with deficiency in nutrient supply [2]. This study shows that low protein diet decreased PI at 120dpc, presumably increasing blood supply and nutrients to the placenta and fetus. This was further enforced by the increased TAMV in these animals. In previous studies, we reported an increase in placental size and function (as measured by higher estrone sulfate (ES)) following a low protein diet during early pregnancy [27,31]. We believe that a similar effect of low protein diet may have occurred in the study reported herein as ES may be rapidly converted to estradiol which in turn may increase uterine blood flow via vasodilation [32],. However, this will need to be studied further in the serum samples collected during the current experiments.

Placental growth from early to midgestation in the cow is much greater than fetal growth and continues to increase exponentially until the last month of pregnancy, when growth slows down [33]. On the contrary, fetal growth during the last trimester is five times that of the placenta, therefore angiogenesis and vascularization must increase to enable sufficient nutrients to be supplied. This pattern of placental and fetal development is similar to the human but dissimilar to the sheep where placental growth plateaus in midgestation [34]. We have previously shown that vasculature in the placenta at term increased following protein restriction in the first trimester [27], similarly Vonnahme *et al*. [35] showed that the placental vasculature increased at term after midgestation restriction followed by re-alimentation.

It is important to note that after nutrient restriction in this study there was no attempt to re-aliment the heifers post dietary restriction. In Expt 1 heifers were individually fed for the entire gestation to restrict such possible variation. The use of only primiparous heifers of restricted breed type, mated to a single bull, reduced possible uterine variation caused by prior pregnancies and variations in fetal demand.

## Conclusions

Protein restriction during the peri-conception period and first trimester altered uterine blood flow in primiparous heifers. Further, uterine blood flow was increased in early pregnancy if the heifer was pregnant to a male rather than a female fetus. Sex specific differences in feto-placental perfusion indices exist in the bovine as previously reported in the human [1] and such indices are susceptible to dietary perturbation. The identification of these effects adds to our knowledge of the physiology of male and female fetuses *in utero*. In accord with a previous report [13] the cardiovascular system of the female fetus was altered by the early *in utero* low protein diet. Fetal organ development has been shown to be affected by changes in maternal nutrition associated with changes in placental hormone production, with such effects having a sex bias. Altered fetal haemodynamics *in utero* may be part of the mechanism involved, making further investigation of the sex specific differences present during fetal life important. This study affords additional insight into the gender specific circulatory differences that exist through gestation.
