# Correction: Gestational Dietary Protein Is Associated with Sex Specific Decrease in Blood Flow, Fetal Heart Growth and Post-Natal Blood Pressure of Progeny

**DOI:** 10.1371/journal.pone.0197942

**Published:** 2018-05-17

**Authors:** Juan H. Hernandez-Medrano, Katrina J. Copping, Andrew Hoare, Wendela Wapanaar, Rosalie Grivell, Tim Kuchel, Giuliana Miguel-Pacheco, I. Caroline McMillen, Raymond J. Rodgers, Viv E. A. Perry

There is an error in the last sentence of the Abstract. The correct sentence is as follows: Protein restriction increased MUA diameter and indices of velocity during late pregnancy, reduced fetal heart weight in the female fetus and increased heart rate at birth, but decreased systolic blood pressure at six months of age.

The images for Figs 2 and 3 are incorrectly switched. The image that appears as Fig 2 should be Fig 3, and the image that appears as Fig 3 should be Fig 2. The figure captions appear in the correct order.

**Fig 2:**
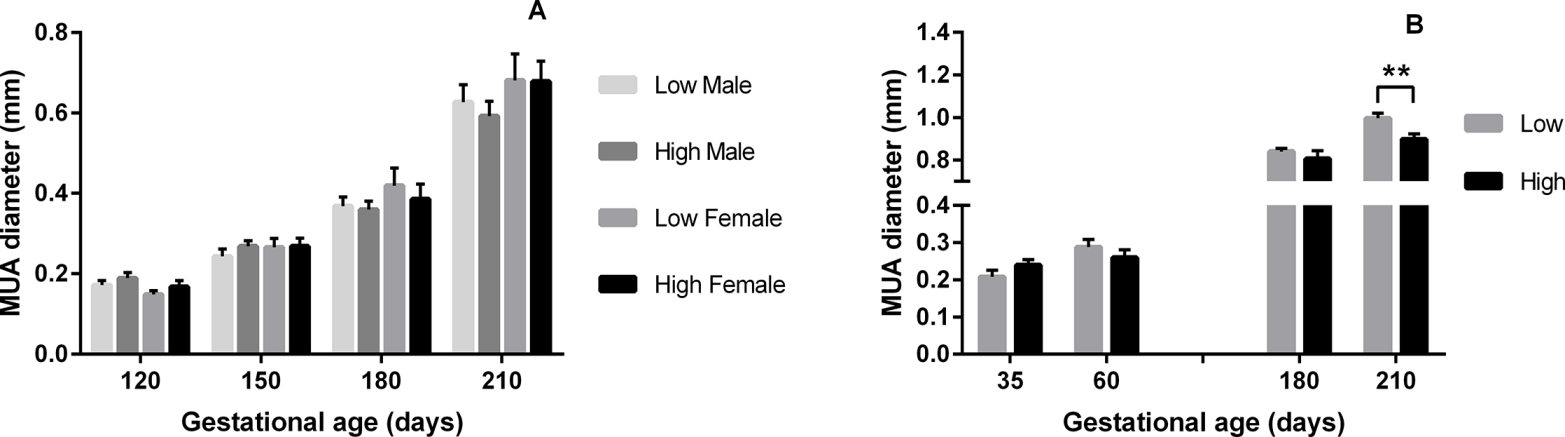
Median Uterine Artery diameter according to PERI and PREconception diet. Mean (±SEM) Median Uterine Artery (MUA) diameter (mm) in heifers carrying male (M) and female (F) fetuses according to A) PERIconception diet Low (7%CP; M n = 20 and F n = 8) and High protein (14%CP; M n = 25 and F n = 11) from 60d prior to conception to 23dpc in Experiment 1 and B) PREconception diet Low (10%CP; M n = 13 and F n = 3) and High protein (18%CP; m n = 7 and F n = 11) from -60d to conception in Experiment 2.; ** denotes P≤0.01.

**Fig 3:**
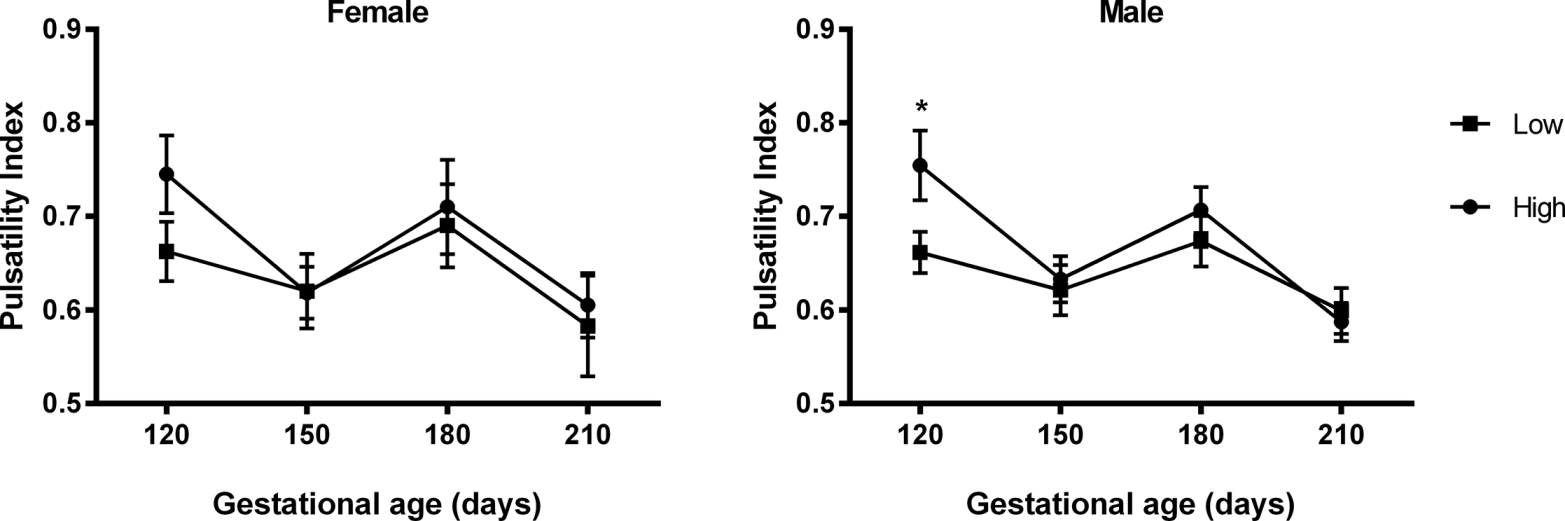
Pulsatility Index according to PERIconception diet. Pulsatility Index (mean ±SEM) by PERIconception diet (■ Low 7%CP and ● High 14%CP) from 60d prior to conception to 23dpc in Experiment 1 in male (Low n = 20 and High n = 25) and female fetuses (Low n = 8 and High n = 11). * denotes P≤0.05.

In the first sentence under the subheading Fetal Heart Weight, the term “PERIconception” is incorrect. The correct term is “POSTimplantation.”

There are multiple errors in the caption for Fig 8. Please see the correct caption below.

**Fig 8:**
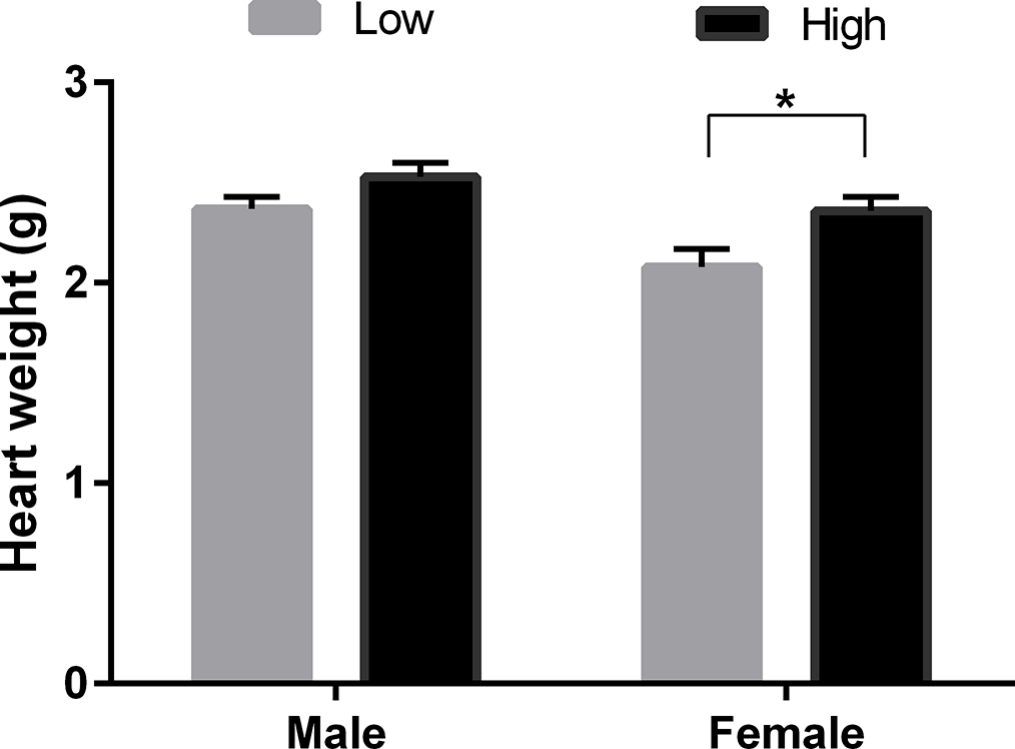
Post-mortem fetal heart weight according to POSTimplantation diet. Mean (±SEM) post-mortem fetal heart weight (g) in male (M) and female (F) 98-day-old fetuses from heifers fed Low (7% CP, M n = 9 and F n = 10) or High (14% CP, M n = 16 and F n = 11) POSTimplantation diet from 23 to 98 days post conception.
